# Supplementary material for: Incidence and Etiology of Postharvest Fungal Diseases Associated with Bulb Rot in Garlic (Alllium sativum) in Spain
Source: Foods. 2021 May 12;10(5):1063. doi: 10.3390/foods10051063 (PMC8151520; doi:10.3390/foods10051063)
Supplement: Supplementary file 1 [file foods-10-01063-s001.zip › foods-1216141-supplementary.pdf]

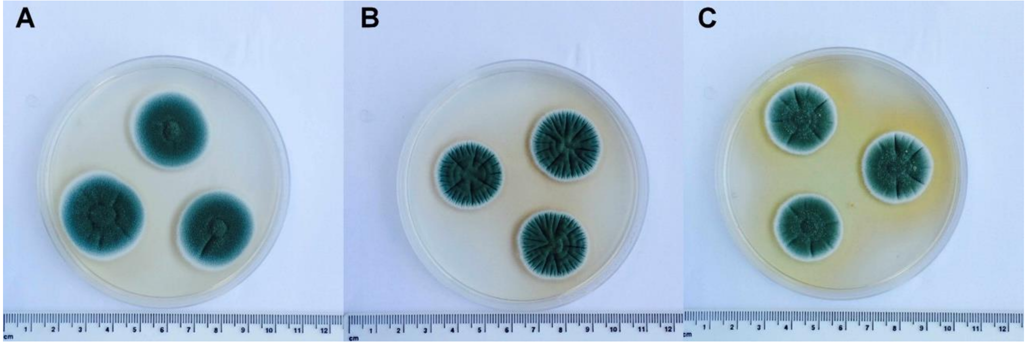

Figure S1. Morphotypes of *Penicillium allii* isolates from garlic in Spain after 7 days at 25 °C in the dark on Czapek yeast agar. (**A**) PA086, morphotype 1; (**B**) PA100, morphotype 2; (**C**) PA112, morphotype 3.

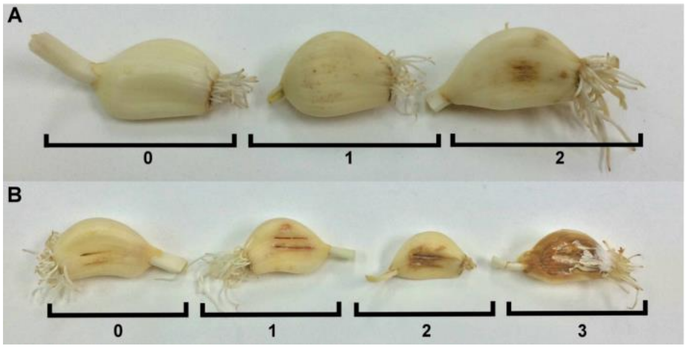

Figure S2. Disease severity index (DSI) scale (0–3) used to evaluate garlic clove rot for the pathogenicity test of *Fusarium* spp. on non-wounded (**A**) and artificially wounded garlic cloves (**B**).
